# Emotional stability is associated with the *MAOA* promoter uVNTR polymorphism in women

**DOI:** 10.1002/brb3.1376

**Published:** 2019-08-25

**Authors:** Ángel Rodríguez‐Ramos, Juan Antonio Moriana, Francisco García‐Torres, Manuel Ruiz‐Rubio

**Affiliations:** ^1^ Department of Genetics University of Córdoba Córdoba Spain; ^2^ Department of Psychology University of Córdoba Córdoba Spain; ^3^ Maimónides Biomedical Research Institute of Córdoba (IMIBIC) Córdoba Spain; ^4^ University Hospital Reina Sofía of Córdoba Córdoba Spain

**Keywords:** anxiety, emotional stability, *MAOA*, neuroticism, phenylthiocarbamide

## Abstract

**Background:**

Neuroticism is associated with low emotional stability, and it is characterized by a tendency to perceive ordinary situations as threatening and difficult to manage. This personality trait has been associated with psychological distress and predicts some mental disorders. Previous studies have shown that women tend to be more neurotic than men and, in general, females have also a higher incidence of anxious and depressive disorders.

**Methods:**

We analyzed in a sample of 99 female university students (from 18 to 26 years old) if emotional stability, measured using the Big Five Questionnaire, was linked to polymorphic variants in candidate genes related to dopaminergic and serotonergic systems, and other personality variables.

**Results:**

We found that emotional stability and its subdimensions are genetically associated with *MAOA*‐uVNTR polymorphism. Thus, women carriers of the 3‐repeat allele (lower *MAO‐A* expression) showed higher levels of emotional stability. No associations were found with other polymorphisms analyzed, including *COMT* Val^158^Met, *5‐HTTLPR*, and *DAT* 3′UTR VNTR. Furthermore, our results showed a negative correlation between emotional stability and depression, state anxiety, and trait anxiety. In fact, *MAOA*‐uVNTR and trait anxiety also explained emotional stability and its subdimensions. We also found that other genetic characteristic, phenylthiocarbamide tasting, explained impulsivity, specifically tasters controlled impulses better than nontasters.

**Conclusion:**

Our results indicate that neuroticism might be regulated by *MAOA* and could be a common factor between different phenotypes, such as aggressive behaviors or personality disorders, observed in women with higher activity genotype who had been exposed to negative environments during childhood. This study could lead to a better understanding of the basis of emotional stability and could lead to future projects for this purpose.

## 1 INTRODUCTION

Neuroticism is a personality trait characterized by a tendency to perceive ordinary situations as threatening and difficult to manage. It correlates with psychological distress and predicts some mental and physical disorders (Slavish et al., 2018). Neuroticism is included in almost all major models of personality traits (Lahey, 2009) and has been associated with emotional instability (even both concepts are used as synonyms). Neurotic individuals tend to worry, experience more negative emotions, and have a poor control of their impulses and desires (Ormel et al., 2013; Servaas et al., 2013). Moreover, persons who score high in neuroticism often feel personally inadequate, and are self‐critical and sensitive to the criticism of others (Lahey, 2009).

Previous studies have shown that women tend to be more neurotic than men (Lynn & Martin, 1997; Weisberg, Deyoung, & Hirsh, 2011). The magnitude of these differences become fewer with age, although a small difference is present even in old age (Chapman, Duberstein, Sörensen, & Lyness, 2007; Soto, John, Gosling, & Potter, 2011). Higher neuroticism scores have been related to some of the most common mental disorders, including mood, anxiety, and substance use disorders (Ormel et al., 2013), and with an unfavorable course of them (Jeuring et al., 2018; Struijs, Lamers, Spinhoven, van der Does, & Penninx, 2018). The higher scores in neuroticism of women respect to men have been related to the fact that women have higher incidence of anxious and depressive disorders than men (Alonso et al., 2004; Riecher‐Rössler, 2017).

Heritability studies using twins have proposed that neuroticism is moderately heritable (~47%) (Boomsma et al., 2018; Docherty et al., 2016; Kim et al., 2017; Power & Pluess, 2015). A recent study has found an association between the dopaminergic system and neuroticism, but only in contexts where there is high climate stress (Fischer, Lee, & Verzijden, 2018). Two genes of this system have been widely associated with certain behaviors and psychiatric conditions: *COMT* and *DAT* (Gatt, Burton, Williams, & Schofield, 2015). COMT is an enzyme involved in degradation of catecholamines such as dopamine, epinephrine, and norepinephrine. The most common genetic variant is a change of valine to methionine (Val^158^Met) in the protein amino acid sequence. Met reduces enzyme activity at only 25% of the Val isoform (Chen et al., 2004; Lotta et al., 1995). On the other hand, DAT is involved in regulating the dopaminergic transmission, reuptaking dopamine from the synaptic cleft to the presynaptic neuron. In the 3′UTR of its coding gene exists a 40 bp variable number of tandem repeats (VNTR) that can present between 3 and 11 copies, being the most common alleles the 9‐ and 10‐repeats forms (Costa, Riedel, Müller, Möller, & Ettinger, 2011). This VNTR affects gene expression, where 9 repeats seem to be associated with higher dopamine reuptaking efficiency than the 10‐repeat allele, although these results are inconsistent with other studies (Del Hoyo et al., 2016; Guo, Roettger, & Shih, 2007).

Nevertheless, actually the most investigated pathway has been the serotonergic system. Current literature suggests that lower synaptic serotonin levels are physiologically responsible of higher neuroticism (Tuominen et al., 2017). 5‐HTT transports the serotonin from the synaptic cleft into the presynaptic neurons. A 44 bp insertion/deletion polymorphism in the promoter region of its coding gene (*5‐HTTLPR*) is associated with changes in the transcriptional activity of the gene, presenting the long allele (L) an increased expression and serotonin reuptake relative to the short variant (S). This polymorphism has been widely studied and has been suggested that has a pleiotropic effect in different mental disorders (Gatt et al., 2015). Human studies linking 5‐HTT and neuroticism have been inconsistent. However, a recent study has shown that neuroticism and 5‐HTT expression are linked in a sex‐dependent manner. In women, higher neuroticism scores seem to be associated with lower 5‐HTT binding in thalamus, whereas in males, higher scores were associated with higher binding (Tuominen et al., 2017).

Enzymes that regulate serotonin levels, such as monoamine oxidases (MAOs), could be also important. MAOs are a family of enzymes that catalyze the oxidative deamination of some neurotransmitters and dietary amines. In human, there are two types of MAOs: MAO‐A and MAO‐B. These protein‐coding genes, *MAOA* and *MAOB*, are located in the X‐chromosome (Grimsby, Chen, Wang, Lan, & Shih, 1991). They have differences in its substrates, tissue‐cell distribution, and other properties. Thus, dopamine, tyramine, and tryptamine are substrates for both MAO‐A and MAO‐B. Nonetheless, MAO‐A preferentially oxidizes the biogenic amines serotonin and norepinephrine (Kalgutkar, Dalvie, Castagnoli, & Taylor, 2001; Shih & Chen, 2004). Brunner syndrome is caused by a *MAOA* mutation that leads to a MAO‐A deficiency and therefore an excess of monoamine neurotransmitters. This rare genetic disorder is characterized by borderline mental retardation and tendency to violence (Brunner, Nelen, Breakefield, Ropers, & Oost, 1993; Hunter, 2010). *MAOA* has a polymorphism 1.2 kb upstream of its coding sequence. This 30 bp VNTR can present 2 (Guo, Ou, Roettger, & Shih, 2008), 3, 3.5, 4 or 5 copies (Sabol, Hu, & Hamer, 1998), affecting the transcriptional expression of the enzyme. Alleles containing 3.5 or 4 repeats are expressed more efficiently than alleles containing either 2, 3 or 5 repeats (Guo et al., 2008; Sabol et al., 1998). Alleles 3 and 4 are the most common forms (Pavlov, Chistiakov, & Chekhonin, 2012). Since *MAOA* is involved in controlling the levels of monoamine neurotransmitters, many studies have focused in genetic associations of *MAOA*‐uVNTR with psychological traits and disorders (Bortolato, Floris, & Shih, 2018; Liu, Huang, Luo, Wu, & Li, 2016). Caspi et al. (2002) provided the first evidence of a gene–environment interaction of the *MAOA* polymorphism that later have been confirmed by several studies (Kim‐Cohen et al., 2006). In this manner, males with low MAO‐A activity genotype who have been exposed to maltreatment tend to show more antisocial and aggressive behaviors. Simultaneously, recent evidence has suggested that this tendency would happen also in women but in those with high MAO‐A activity genotype (Aslund et al., 2011; Byrd & Manuck, 2014; McGrath et al., 2012; Prom‐Wormley et al., 2009; Sjöberg et al., 2007; Verhoeven et al., 2012; Wakschlag et al., 2010). *MAOA* has been related to other mental health problems in addition to antisocial behaviors. Recent studies have suggested that could exist a common factor for all these phenotypes, and that could be emotion regulation (Nilsson, Åslund, Comasco, & Oreland, 2018). Then, women with high MAO‐A activity genotype who had experienced childhood maltreatment would present higher levels of emotional reactivity that could predict some pathologies, with the reverse pattern in males. Thereby, *MAOA* x maltreatment would regulate indirectly through emotional reactivity a higher risk of some pathologies rather than through a direct way (Byrd et al., 2019).

Although aggressiveness and impulsivity are two separated constructs, they are closely related (García‐Forero, Gallardo‐Pujol, Maydeu‐Olivares, & Andrés‐Pueyo, 2009). In the Big Five Questionnaire (Caprara, Barbaranelli, Borgogni, & Perugini, 1993), the dimension Emotional Stability‐Neuroticism is subdivided into emotion control and impulse control. So, this construct is highly related to impulsivity, which is a multidimensional neuropsychological construct that can be defined as “a predisposition toward rapid, unplanned reactions to internal or external stimuli without regard to the negative consequences of these reactions to the impulsive individual or to others” (Moeller, Barratt, Dougherty, Schmitz, & Swann, 2001). Despite any person can present impulsivity, it is more probable to be present in people with certain psychiatric disorders, such as ADHD, mania, substance abuse/dependence, and some personality disorders (Kulacaoglu & Kose, 2017; Moeller et al., 2001).

Some psychiatric conditions as anhedonia (Thomas, Al‐Mesaabi, Bahusain, & Mutawa, 2014) and different personality traits (Kimmel & Lester, 1987; Mascie‐Taylor, McManus, MacLarnon, & Lanigan, 1983; Very & Iacono, 1968) have been related to phenylthiocarbamide (PTC) tasting. PTC is an organic compound that either tastes bitter or is tasteless. This capacity to taste the PTC appears to depend on a simple dominant Mendelian gene (**t**asters > non**t**asters), although some reports suggest a more complex inheritance and expression of this trait (Guo & Reed, 2001; Keller & Adise, 2016). This capacity has been also related to numerous conditions such as food selection and obesity (Veluswami et al., 2015), smoking (Risso et al., 2016), or disorders as schizophrenia (Moberg et al., 2007).

Since *MAOA*‐uVNTR x maltreatment have been associated with emotional reactivity, we proposed that *MAOA* could be related in some way with emotional stability–neuroticism personality trait. Likewise, various studies have investigated whether there exists a genetic association between PTC tasting, *COMT*, *5‐HTT*, and *DAT* polymorphisms with this trait, and several discrepancies have been detected. In the present study, we attempted to extend in an integrative way the knowledge of how neuroticism, emotion control, and impulse control are affected by these variables in women.

## 2 MATERIALS AND METHODS

### 2.1 Participants

The initial sample was *N* = 132, students of the University of Córdoba (Spain). However, some subjects of the study were dismissed due to missing data, unwillingness to participate, or age higher than the established for the study. Finally, ninety‐nine healthy women between 18 and 26 years old (mean = 20.89 years; *SD* = 1.846 years) took part in the current research. The participants were randomly selected among the students of two university centers. All procedures were carried out with the understanding and written consent of each subject. Once informed consent was provided, participants were assigned with an individual code and completed an anonymous self‐report survey. They also permitted to collect buccal swabs for genomic DNA. The study was approved by “Comité de Ética de la Investigación de Córdoba, Hospital Universitario Reina Sofía, Córdoba (Spain)”.

### 2.2 Measures

#### 2.2.1 Big Five Questionnaire (BFQ)

The Spanish version of the BFQ (Caprara, Barbaranelli, & Borgogni, 1995) consists of 132 multiple‐choice items of a 5‐points Likert scale. This questionnaire is a personality test based on the Five‐Factor Model that identifies five fundamental dimensions in the human personality (Energy, Friendliness, Conscientiousness, Emotional Stability, and Openness). In the BFQ, each dimension is subdivided into two subdimensions. These dimensions and its subdimensions are measured in a continuum where an individual might be anywhere between the two extremes of each one. The BFQ also includes a Distortion scale with the purpose of identifying altered profiles. In the present study, we will only mention the dimension emotional stability–neuroticism that is subdivided into impulse control and emotion control.

#### 2.2.2 State‐Trait Anxiety Inventory (STAI)

The STAI is a psychological questionnaire commonly used to estimate state and trait anxiety (Spielberger, Gorsuch, & Lushene, 1970). The Spanish version of this inventory consists of 20 items for assessing state anxiety and 20 for trait. In each item, subjects indicate in which extend they agree or disagree with the statements with a 4‐point Likert scale as “very much”, “likely”, “not so much,” or “not at all”, where the response scale ranges from 0 to 3 points (Buela‐Casal, Guillén‐Riquelme, & Seisdedos Cubero, 2011). Higher scores indicate greater anxiety. State anxiety reflects a transitory emotional state or “right now” condition. However, trait anxiety represents stable individual differences in the tendency to anxiety and refers to a prone to respond with “general” anxiety.

#### 2.2.3 Beck's Depression Inventory (BDI‐II)

The Spanish version of BDI‐II is a 21‐question multiple‐choice self‐report inventory to measure symptoms of depression, in which each item was rated on a scale from 0 to 3 (Beck, Steer, & Brown, 2011). The total score was calculated by summing the score of the 21 items and oscillates between 0 and 63 points, where higher scores indicate greater symptom severity. Different cutoff ranges have been established: 0–13 minimal depression, 14–19 mild depression, 20–28 moderate depression, and 29–63 severe depression (Beck, Steer, & Brown, 1996).

### 2.3 Genotyping

Genomic DNA was extracted and purified from buccal swabs through the HigherPurity™ Buccal Swab Genomic DNA Extraction Kit (Canvax Biotech S.L.). Different polymorphisms were genotyped through polymerase chain reaction (PCR) amplification (Canvax Biotech S.L.) in a final volume of 10 μl, containing: 1 μl of about 100 ng DNA, 1 μl of 10x PCR Buffer, 1 μl of 25 mM MgCl_2_, 1 μl of 8 mM dNTP, 3 μl of 10x GC‐enhancer, 2 μl of 10x Y‐enhancer, 0.25 μl of forward primer (15 pmol/μl), 0.25 μl of forward reverse (15 pmol/μl), 0.25 μl of Mili‐Q water, and 0.25 μl Taq DNA polymerase (5 U/μl).

#### 2.3.1 Monoamine oxidase A (MAOA)

For genotyping *MAOA*‐uVNTR polymorphism, we used the following primers based on Sabol et al. (1998): forward 5′‐ACA GCC TGA CCG TGG AGA AG‐3′ and reverse 5′‐GAA CGG ACG CTC CAT TCG GA‐3′. PCR conditions were an initial denaturalization of 8 min at 95°C, 40 cycles of 30 s at 94°C, 30 s at 58.2°C, and 1 min at 72°C; followed by a final elongation of 8 min at 72°C. PCR products were separated and visualized using 2.5% agarose gels with ethidium bromide. This protocol is a modification of McGrath et al. (2012).

Alleles with 3 repeats were categorized as “low” activity, while those with 4 repeats were categorized as “high” activity. Genotype frequencies of the genetic polymorphisms in the sample are collected in Table 1. Genotype frequencies of our sample are similar to previously described (Table 1), so we assumed that it was representative of the population. The population was in Hardy–Weinberg equilibrium, *χ* ^2^(1, *N* = 99)=1.79, *p *< .05. Since *MAOA* gene is located in the X‐chromosome, it is easy to determine whether they present high or low MAOA activity in men. However, it is not clear if in women the *MAOA* gene of the second chromosome is silenced or not. Some studies have shown that *MAOA* expression is silenced on the inactive X‐chromosome in women (Nordquist & Oreland, 2006; Stabellini, Vasques, de Mello, Hernandes, & Pereira, 2009). Others, even though do not find a complete X‐inactivation of the *MAOA*, took to indicate the existence of an alternative process of dosage compensation in females highly variable among individuals (Pinsonneault, Papp, & Sadée, 2006). Taking this into account, if X‐inactivation/regulation is sufficiently random across cells, in heterozygous this would predict an intermediate response generally lower than the homozygous for 4‐repeat allele as other have previously observed (Meyer‐Lindenberg et al., 2006). Since it is not completely understood, we preferred to take both options into consideration. Likewise, since the 4‐repeat allele is the most common allele, we had a little percentage of women homozygous for 3 repeats. For these reasons, we considered to create only two groups, one with the highest expression (homozygous for 4‐repeat allele) and other with lower expression (heterozygous and homozygous for 3‐repeat allele).

**Table 1:** Distribution of the genetic polymorphisms studied in the sample

Variable	*n*	%	%^a^
*5‐HTT*			Arias et al. (2012)
L/L	24	24.2	27.4
L/S	47	47.5	48
S/S	28	28.3	24.6
*DAT*			Sáiz et al. (2010)
9R/9R	8	8.1	10.89
9R/10R	47	47.5	42.9
10R/10R	41	41.4	42.25
10R/11R	3	3.0	Ns
*MAOA*			Rivera et al. (2009)
3R/3R	12	12.1	17
3R/4R	37	37.4	39
4R/4R	50	50.5	42
*COMT*			Sánchez‐Morán et al. (2018)
Val/Val	27	27.3	25.0
Val/Met	49	49.5	50.0
Met/Met	23	23.2	25.0

a Proportions previously described in Spanish populations.

#### 2.3.2 Catechol‐O‐methyltransferase (COMT)

We genotyped the *COMT* Val^158^Met polymorphism using the following primers: forward 5′‐ACT GTG GCT ACT CAG CTG TG‐3′ and reverse 5′‐CCT TTT TCC AGG TCT GAC AA‐3′. As described by Bishop, Fossella, Croucher, and Duncan (2008), PCR conditions were an initial denaturalization of 5 min at 94°C, 12 cycles of 30 s at 94°C, 45 s at 58°C, and 35 s at 72°C; followed by 28 cycles of 30 s at 94°C, 45 s at 50°C, and 30 s at 72°C. The final point was an elongation of 5 min at 72°C. PCR products were digested using the restriction enzyme *NlaIII*, also known as *Hin1II* (Thermo Scientific). After digested, samples were separated in a 3% agarose gel with ethidium bromide. The population was in Hardy–Weinberg equilibrium, *χ* ^2^(1, *N* = 99) = 0, *p *< .05.

#### 2.3.3 Dopamine transporter (DAT)

We genotyped *DAT* 3′UTR VNTR using the following primers based on Vandenbergh et al. (1992): forward 5′‐ TGC GGT GTA GGG AAC GGC CTGA GA‐3′ and reverse 5′‐GTG TGG TCT GCA GGC TGC CTG CAT‐3′. PCR conditions were an initial denaturalization of 5 min at 95°C, 40 cycles of 30 s at 95°C, 30 s at 68°C, and 90 s at 72°C and a final elongation of 5 min at 72°C. PCR products were separated in a 2% agarose gel with ethidium bromide. The population was in Hardy–Weinberg equilibrium, *χ* ^2^(1, *N* = 99) = 2.70, *p *< .05.

#### 2.3.4 Serotonin transporter (5‐HTT)

We genotyped *5‐HTTLPR* polymorphism using the following primers based on Lesch et al. (1996): forward 5′‐GGC GTT GCC GCT CTG AAT GC‐3′ and reverse 5′‐ GAG GGA CTG AGC TGG ACA ACC AC‐3′. PCR conditions were an initial denaturalization of 5 min at 95°C, 40 cycles of 30 s at 95°C, 30 s at 68°C, and 90 s at 72°C and a final elongation of 5 min at 72°C. PCR products were separated in a 2% agarose gel with ethidium bromide. The population was in Hardy–Weinberg equilibrium, *χ* ^2^(1, *N* = 99) = 0.161, *p *< .05.

### 2.4 Phenylthiocarbamide (PTC) taste test strips

The PTC test strips were pre‐prepared in the laboratory using rectangular fragments of filter paper impregnate with a suprathreshold PTC solution. Stock solutions were freshly prepared containing 2.60 g of the recrystallized material per liter (Lugg & Whyte, 1955). The subjects were asked to hold a filter paper without the solution (control), and later with the solution (test), in her mouth for 5–10 s and tell if they taste a bitter flavor or not in the second one. Depending on it, they were grouped in “tasters” and “nontasters,” respectively.

### 2.5 Statistical analysis

Statistical analysis was carried out using IBM SPSS Statistics 20. Groups were compared using independent ANOVA test on genetic polymorphisms, PTC tasting, and sociodemographic variables. Pearson correlation coefficient was used to calculate bivariate correlations among the different quantitative variables analyzed. Finally, three multiple linear regressions were carried out using the stepwise method in order to stablish the explanatory power of all the independent variables measured herein (state and trait anxiety, depression, *MAOA*, *COMT*, *5‐HTT* and *DAT* polymorphisms, and PTC tasting) over the selected dependent variables (emotional stability and its subdimensions). Results were accepted as significant with a confidence level of 95%.

## 3 RESULTS

### 3.1 Emotional Stability negative correlates with Anxiety and Depression levels

Mean scores of the self‐report questionnaires have been brought together in Table 2; ANOVA and correlation analysis results have been collected in Tables 3 and 4, respectively. As could be expected, we found a negative correlation between emotional stability and state and trait anxiety. There was also a negative correlation between emotional stability and depression. These negative correlations existed even with the two subdimensions of the emotional stability.

**Table 2:** Mean score and standard deviation of self‐report variables

Variable	*n*	%	Mean	*SD*
Anxiety (STAI)				
State	99		14.89	9.335
Trait	99		21.48	9.693
Depression (BDI)	99		6.27	5.776
BFQ				
Emotional stability	99		51.47	10.205
Emotion control	99		51.17	9.316
Impulse control	99		51.48	10.948
PTC				
Taster	74	74.7		
Nontaster	25	25.3		

Abbreviations: BDI, Beck's Depression Inventory; BFQ, Big Five Questionnaire; PTC, phenylthiocarbamide; STAI, State‐Trait Anxiety Inventory.

**Table 3:** Statistical results of ANOVA analysis

Variable 1	Variable 2	*F* (1,99)	*p* value
Emotional stability	*MAOA*	6.925	.010
	*COMT*	1.218	.299
	*5‐HTT*	0.248	.781
	*DAT*	0.621	.603
	Phenylthiocarbamide	1.499	.224
Impulse control	*MAOA*	10.056	.002
	*COMT*	1.377	.256
	*5‐HTT*	0.118	.888
	*DAT*	0.671	.572
	Phenylthiocarbamide	6.118	.015
Emotion control	*MAOA*	2.780	.099
	*COMT*	0.880	.417
	*5‐HTT*	0.564	.571
	*DAT*	0.621	.603
	Phenylthiocarbamide	0.065	.800

**Table 4:** Statistical results of correlation analysis

Variable 1	Variable 2	*r* (97)	*p* value
Emotional stability	State anxiety	−.469	.001
	Trait anxiety	−.674	.001
	Depression	−.523	.001
Impulse control	State anxiety	−.343	.001
	Trait anxiety	−.527	.001
	Depression	−.387	.001
Emotion control	State anxiety	−.498	.001
	Trait anxiety	−.699	.001
	Depression	−.566	.001

### 3.2 Association of low‐activity MAO‐A allelic variant with emotional stability

We also found a genetic association between *MAOA*‐uVNTR and neuroticism in females. Women carriers of the 3‐repeat allele showed higher levels of emotional stability, and then lower neuroticism, than those noncarriers of this allele. Referring to the subdimensions of emotional stability, carriers of the 3‐repeat allele also presented higher impulse control. However, there was no significant difference in emotion control, but it showed a tendency toward significance.

### 3.3 No association between COMT, 5‐HTT, and DAT polymorphisms and emotional stability

We checked out also if there was a genetic association between *COMT*, *5‐HTT*, and *DAT* polymorphisms and neuroticism or its subdimensions, however, we did not find any significant result.

### 3.4 Phenylthiocarbamide tasting capacity is linked to a better impulse control

The frequency of phenylthiocarbamide tasters in the sample (Table 2) was similar to previous studies carried out in Spanish population (Guo & Reed, 2001; Pons, 1955). We found that phenylthiocarbamide tasters controlled better their impulses than nontasters. However, no significant differences were found in emotion control or emotional stability.

### 3.5 Regression models of emotional stability, impulse control, and emotion control

Furthermore, we evaluated the explanatory power of the variables measured herein over emotional stability, impulse control, and emotion control. For this purpose, we first included all the variables in the regression analysis, and later, we were discarding those that were not significant until only significant variables were included in the model.

The results showed that variables included in the different models were significant (Table 5). In our model for emotional stability, trait anxiety and *MAOA* polymorphism explained the 51% of the variance observed in our sample (*R* ^2^ = .512, *F*(2,99) = 52.453, *p *< .001). In the case of impulse control, the 38.6% of the variance was explained by trait anxiety, *MAOA* polymorphism, and PTC tasting (*R* ^2^ = .386, *F*(3,99) = 21.503, *p *< .001). Finally, the 50.7% of the variance observed in emotion control was explained by trait anxiety and *MAOA* polymorphism (*R* ^2^ = .507, *F*(2,99) = 51.414, *p *< .001). Residual plots of the different models have been included in Figure S1.

**Table 5:** Regression models of emotional stability, impulse control, and emotion control

Predictor variable	*B*	*SE B*	*β*	*t*	*p*
Model for emotional stability					
*Trait anxiety*	−0.711	0.074	−.675	−9.566	<.001
*MAOA* (high MAO‐A activity genotype)	−5.307	1.433	−.261	−3.704	<.001
Model for impulse control					
*Trait anxiety*	−0.594	0.089	−.526	−6.638	<.001
*MAOA* (high MAO‐A activity genotype)	−5.936	1.762	−.272	−3.369	<.001
*PTC* (tasters)	4.499	2.028	.179	2.219	.029
Model for emotion control					
*Trait anxiety*	−0.672	0.068	−.700	−9.863	<.001
*MAOA* (high MAO‐A activity genotype)	−3.156	1.315	−.170	−2.400	.018

## 4 DISCUSSION

The present study was designed to examine the role that several genetic polymorphisms related to the dopaminergic and serotonergic pathways play in neuroticism. We have focused in women since they have higher incidence of mental disorders than men, especially anxious and depressive disorders (Alonso et al., 2004; Riecher‐Rössler, 2017). Several studies have reported that women often show higher scores in neuroticism than men (Lynn & Martin, 1997; Weisberg et al., 2011). At the same time, higher neuroticism scores have been related to some affective disorders (Ormel et al., 2013) and an unfavorable course of them (Jeuring et al., 2018; Struijs et al., 2018). These data agree with the results present herein that show a negative correlation between emotional stability (and its subdimensions) and anxiety and depression levels. Thereby, maybe sex differences in neuroticism might explain the fact that women suffer more from this type of mental disorders (Kendler & Gardner, 2014).

### 4.1 MAO‐A and the serotonergic system: Implications in emotional stability

Twin studies have suggested that neuroticism has a moderate genetic heritability (Boomsma et al., 2018; Power & Pluess, 2015). In this context, genes related to dopaminergic and serotonergic systems are good candidates to explain part of the genetic basis of this personality trait. In the present study, we have found a significant genetic association between neuroticism and *MAOA* 30 bp VNTR polymorphism, which protein is involved in degradation of serotonin and dopamine. Specifically, we have found that healthy women with higher MAO‐A activity genotype show higher levels of neuroticism than those with lower activity.

Referring to the serotonergic system, the current literature shows an increase in frontolimbic binding of the 5‐HT_2A_ (Frokjaer et al., 2008) and a reduction of 5‐HT_1A_ binding in cortical and subcortical regions in people with high neuroticism scores (Hirvonen, Tuominen, Någren, & Hietala, 2015). A recent study has shown that neuroticism and 5‐HTT expression are linked in a sex‐dependent manner, where higher neuroticism scores seem to be associated with lower 5‐HTT binding in thalamus in women (Tuominen et al., 2017). Cell surface expression of 5‐HTT quickly responds to dynamic serotonin concentrations, generally being the expression of 5‐HTT parallel to serotonin levels (Ramamoorthy, Giovanetti, Qian, & Blakely, 1998), that could be interpreted as women have lower levels of serotonin in the thalamus. This might explain why did not find a genetic association between 5‐HTT and neuroticism in our study since we only carried out it in women. So, in summary, the current literature suggests that lower synaptic serotonin levels are the physiological basis of higher neuroticism in women. The fact that our results show higher levels of neuroticism in women with higher MAO‐A activity genotype agrees with this hypothesis, considering that it is the main enzyme involved in degrading serotonin. Thus, women with higher MAO‐A activity will degrade more serotonin and then probably have lower serotonin levels.

Results shown herein are comparable to Yu et al. (2005), who reported higher harm avoidance in females with higher MAO‐A activity, which is related to depression and anxiety disorders. Eley et al. (2003) find a genetic association between high MAO‐A activity and neuroticism in men, but not in women. Otherwise, our results contrast with several studies that have found no association between *MAOA* and neuroticism (Garpenstrand et al., 2002; Pełka‐Wysiecka et al., 2012; Tochigi et al., 2006). These discrepancies might be due to the use of different test to measure neuroticism. In fact, our study is the first report of a genetic association between the BFQ and *MAOA*‐uVNTR. Another reasonable explanation could be the different range of age of the sample, in other investigations the mean age of the sample was higher than in our study (a mean of around 40 years old), and some of them present a wide range of age between participants or do not include it. As reported by Soto et al. (2011), neuroticism in women shows positive trends into adolescence, flat trends through emerging adulthood, and then a negative trend across early adulthood and middle age. So, it is likely that age might influence on finding a relationship between the variables here studied and should be considered.

### 4.2 MAO‐A, aggression, and impulsivity

We also found a genetic association between MAO‐A and impulse control. While males with low MAO‐A activity who have been exposed to maltreatment tend to show more antisocial and aggressive behaviors (Caspi et al., 2002), in women this tendency would happen but in those with high MAO‐A activity (Byrd & Manuck, 2014). Likewise, males with low MAO‐A activity present an increasing activity of the amygdala and the hippocampus with the level of stress during childhood, while males with high MAO‐A activity exhibit a decreasing response. The opposite occurs in women (Holz et al., 2016). Consequently, MAO‐A gene–environment interaction seems to be sex‐dependent, at least in the neural circuitry of aggression.

It has been described that excessive reactivity in the amygdala coupled with inadequate prefrontal regulation contribute to increase the likelihood of aggressive behavior (Pavlov et al., 2012; Siever, 2008). Particularly, an increased amygdala activity has been linked to impulsive aggression and not to callous‐unemotional aggression. This kind of aggression has previously been associated with *MAOA* (Meyer‐Lindenberg et al., 2006). Although impulsivity and aggressiveness are two separated constructs, they are closely related (García‐Forero et al., 2009). In this way, our results show that women with high MAO‐A activity would control less their impulses and hence would be more impulsive than those with low MAO‐A activity. This higher impulsivity might predict, even more, the fact that they also present more aggressive behaviors (impulsive aggression) and other risk behaviors related to mental disorders when they are exposure to childhood life stress. Then, women with high MAO‐A activity genotype would present higher neuroticism and impulsivity and, in a negative environment, are more likely to present aggressive behaviors, personality disorders, and other phenotypes. Neurotic people tend to present more emotional reactivity (Lahey, 2009). Therefore, these results would agree with Byrd et al. (2019) who found that high MAO‐A activity genotype would be related to aggressive behaviors and other disorders through an indirect pathway with emotional reactivity as an intermediary instead of through a direct pathway. Thus, we can speculate that neuroticism regulated by *MAOA* might be a common factor between the different phenotypes observed in women who had the high MAO‐A activity genotype as proposed by Nilsson et al. (2018).

### 4.3 Dopaminergic system and neuroticism

In this study, we also try to find a genetic association with other polymorphisms in genes related to the dopaminergic system. Particularly, *COMT* and *DAT* are involved in degrading dopamine and reuptaking dopamine from the synaptic cleft to the presynaptic neurons, respectively.

In accordance with our results, several studies did not find an association between the *COMT* Val^158^Met polymorphism and neuroticism in the revised version of the Eysenck Personality Questionnaire in large samples (Henderson et al., 2000; Olsson et al., 2005; Pap et al., 2012; Urata et al., 2007; Wray et al., 2008). Other studies only found a tendency toward significance (Hatzimanolis et al., 2013; Hoth et al., 2006; Pełka‐Wysiecka et al., 2012). Otherwise, other studies have found a significant association between the *COMT* polymorphism and differences in neuroticism in a sex‐dependent manner on the NEO‐FFI in healthy people (Eley et al., 2003; Hettema, Chen, Sun, & Brown, 2015; Stein, Fallin, Schork, & Gelernter, 2005). Taking all together, this information seems to indicate that *COMT* Val^158^Met polymorphism is not directly associated with neuroticism or if it is, it would explain just a little percentage of neuroticism. One supposed explanation could be that the main mechanism involved in neuroticism is the serotonergic system, and serotonin is not degraded by COMT.

Some studies have been carried out associating *DAT* 3′UTR VNTR polymorphism and neuroticism, and most of them did not find significant results (Chmielowiec et al., 2018; Hünnerkopf, Strobel, Gutknecht, Brocke, & Lesch, 2007; Kazantseva, Gaĭsina, Malykh, & Khusnutdinova, 2009; Kazantseva, Gaysina, Malykh, & Khusnutdinova, 2011). However, a recent meta‐analysis has suggested that the dopaminergic system is related to personality trait differences in neuroticism only in contexts where there is a high climatic stress (Fischer et al., 2018). Although they consider Spain as a place with medium climate demands, we did not find a genetic association.

In summary, we found no genetic association between neuroticism and these two genes of the dopaminergic system. It is likely that since personality is a complex construct and it is influenced by both genes and environment, many different genes with small effect would contribute to its expression, and our sample is not big enough to detect its effect.

### 4.4 PTC might be associated with impulse control

Phenylthiocarbamide is an organic compound that either tastes bitter or is tasteless. Previous studies have tried to find an association between PTC tasting and mental conditions, but just a few of them are related to personality. Very and Iacono (1968) found that nontasters scored significantly higher on six MMPI clinical scales (D, Hy, Pd, Pa, Pt, and Sc scales), while tasters only scored higher on the Ma scale. They interpreted these results as a general maladjustment of the nontasters and, hence, a higher susceptibility to organic problems. In the present study, we have found that nontasters control worse their impulses. So, our results seem to agree with this previous study. On the other hand, Mascie‐Taylor et al. (1983) found that nontasters tend to be more placid, relaxed, and practical in comparison with the tasters using Cattell's Sixteen Personality Factor Questionnaire. Likewise, Kimmel and Lester (1987) found no difference between tasters and nontasters in the psychological traits of the Eysenck Personality Inventory. In these mentioned reports, different questionnaires were used. This could explain the discrepancies between the results found in them, suggesting that further investigation is required.

### 4.5 Explanatory power of the regression models

Finally, we carried out a linear regression to check out if the variables studied herein would explain emotional stability or any of it subdimensions. We found that trait anxiety highly explains emotional stability, while state anxiety and depression does not. This is not surprising since Ormel, Rosmalen, and Farmer (2004) found a strong correlation between the stable component of anxiety and neuroticism. The same was observed with its subdimensions, impulse control, and emotion control. The *MAOA*‐uVNTR polymorphism also explains these three variables, while PTC tasting only explains impulse control.

Our models would explain 51% of the variance in emotional stability, 38.6% in impulse control, and 50.7% in emotion control. Although all the models did not explain all the variance in these variables, it is not unexpected since these variables are complex constructs that depend on genetics and also the environment. We could expect that including other variables as exposition or not to a stressful environment would change the model, probably explaining a higher percentage of the variance observed.

### 4.6 Limitations and future directions

In the present research, we have studied how emotional stability is affected by personality, genetics, and other phenotypic variables such as being taster of PTC in women. It is the first time that these variables have been checked out altogether trying to explain emotional stability, impulse control, and emotion control evaluated through the BFQ. These are complex constructs that depend on multiple factors, and it would be interesting to consider other variables in family members and environmental characteristics during childhood.

As main limitation of our study, we have to mention the sample size that is relatively low for this kind of study. However, some authors mentioned that largest studies are less thoroughly performed than small studies, and therefore, it is also important to taking small studies into account for meta‐analysis (Moffitt & Caspi, 2014). Duncan and Keller (2011) argued that there is a strong publication bias toward positive findings in novel GxE reports and that probably many more tests had been carried out than reported in the literature because of that. They also found that normally replication studies are more realistic of the true rate of positive GxE findings than do novel studies. In this sense, although our study is a novel research, it is based on previous reports but with a new integrative perspective and we also showed negative results obtained for the dopaminergic system genes. Likewise, in our study we carried out a large number of analysis, so it is likely that appears a multiple testing problem. This problem is frequent in genomic studies and other fields related to biology, circumstance that must be taken into account when interpreting the results obtained. Thus, although our results are in line with previous reports, we cannot discard the possibility of type I error. In any case, this study could give rise to future projects that help to elucidate how emotional stability is influenced by both genes and environment and which mechanisms are involved in it, starting with the serotonergic system.

## 5 CONCLUSIONS

In the present study, we have found that women with higher MAO‐A activity genotype (homozygous for 4‐repeat allele) showed lower emotional stability, and then higher neuroticism, and impulse control than those with lower MAO‐A activity genotype (homozygous for 3‐repeat allele and heterozygous). Previous studies have shown that women with higher MAO‐A activity genotype who had been exposed to negative environments during childhood presented higher risk of presenting aggressive behavior and some mental disorders as personality disorders. We propose that in women, neuroticism might be regulated by *MAOA* and could be a common factor between all these phenotypes observed in women with higher MAO‐A activity genotype. We also found that depression, state anxiety, and trait anxiety were highly associated with emotional stability and its subdimensions. On the other hand, PTC tasters controlled better their impulses than nontasters and no associations were found with other polymorphisms analyzed, including *COMT* Val^158^Met, *5‐HTTLPR*, and *DAT* 3′UTR VNTR. Finally, from all these variables, only *MAOA*‐uVNTR and trait anxiety explained some of the variance observed in emotional stability, impulse control, and emotion control, while PTC tasting also explained a percentage of the variance observed in impulse control.

## CONFLICT OF INTEREST

None declared.

## Data Availability

Data available on request due to privacy/ethical restrictions.
