# Supplementary material for: Emotional stability is associated with the MAOA promoter uVNTR polymorphism in women
Source: Brain Behav. 2019 Aug 25;9(9):e01376. doi: 10.1002/brb3.1376 (PMC6749489; doi:10.1002/brb3.1376)

Supplementary Figure S1.
Residual plots of the multiple regression models.

Model for emotional stability

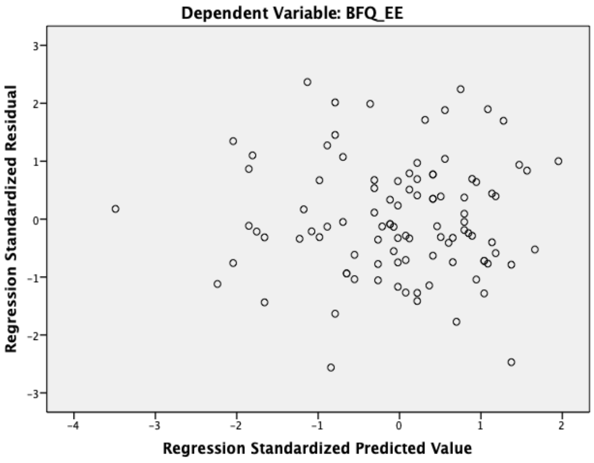

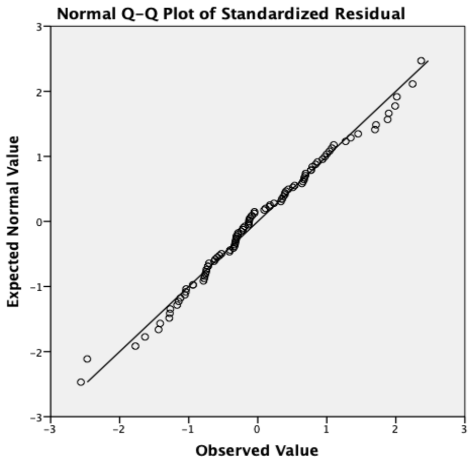

Model for impulse control

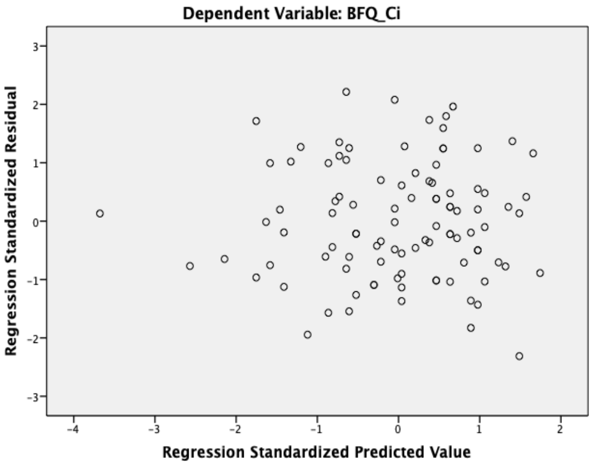

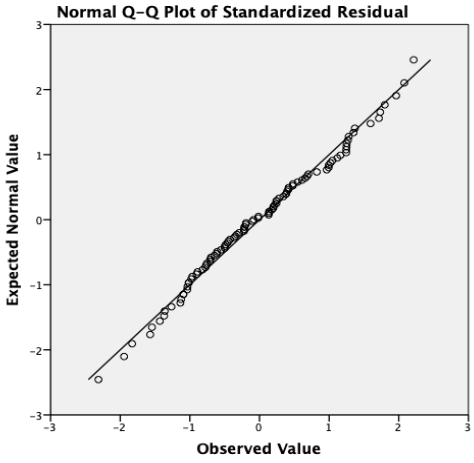

Model for emotion control

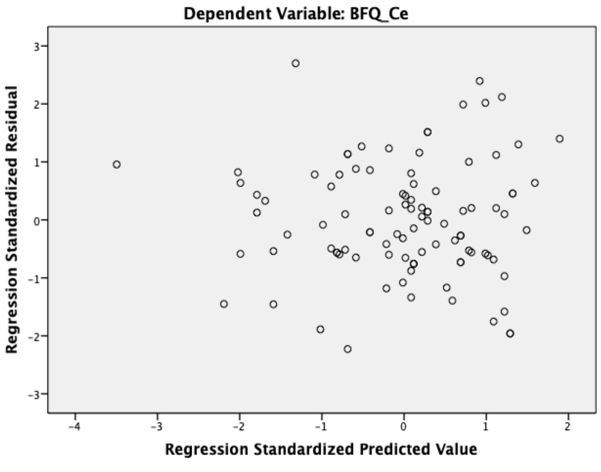

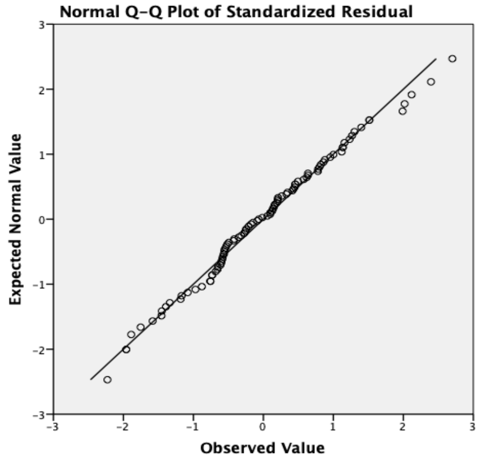

Supplement: Supplementary file 1 [file BRB3-9-e01376-s001.pdf]
